# Effectiveness of Advanced Platelet-Rich Fibrin on Postoperative Sequelae for Impacted Mandibular Third Molar Surgery: A Prospective Study

**DOI:** 10.7759/cureus.52297

**Published:** 2024-01-15

**Authors:** Lavanya Mathialagan Kalai Selvam, Arun M, Saravanan Lakshmanan, Santhosh P Kumar

**Affiliations:** 1 Oral and Maxillofacial Surgery, Saveetha Dental College and Hospitals, Saveetha Institute of Medical and Technical Sciences, Saveetha University, Chennai, IND

**Keywords:** platelet-rich fibrin, mandibular third molar surgery, platelet concentrates, novel technique, innovative, ­wound healing

## Abstract

Introduction

Impacted third molar extraction is frequently removed surgically by maxillofacial surgeons, which is mostly associated with postoperative sequelae like pain and swelling. It is essential to minimize the complications and enhance wound healing in the extracted socket of the third molar. Hence, this study aimed to assess the efficiency of advanced platelet-rich fibrin (A-PRF) in wound healing and reducing pain after surgical extraction of the impacted mandibular third molar (IMTM).

Materials and methods

Thirty healthy patients who have been diagnosed with Pell and Gregory class II IMTM were included in this study. In the study group, which comprises 15 patients, extraction sockets were filled with A-PRF extract. In the control group, no material was placed in the extraction sockets. The pain was assessed preoperative and on the third and seventh postoperative days using a visual analog scale (VAS). Wound healing was assessed on the third and seventh postoperative days using a modified laundry scale. SPSS for Windows was used for data analysis. Categorical data was compared between the groups using the Chi-square test. P-value less than 0.05 was considered as statistically significant.

Results

The study population's mean age was 25.67 ± 2.4 years. Nineteen patients were male, and 11 patients were female. Differences in mean pain scores between the groups were not statistically significant both on the third postoperative day (p=0.59) and the seventh postoperative day (p=0.33). During the seventh day postoperative day, the study group exhibited better wound healing compared to the control group and the results were statistically significant (p=0.01).

Conclusion

A-PRF is a simple and effective method of reducing postoperative sequela by promoting wound healing after surgical extraction of IMTM. It has the advantage of less chance of allergic and anaphylactic reactions, unlike their predecessor platelet concentrates.

## Introduction

Impacted teeth are completely or partially unerupted and are positioned against another tooth, bone, or soft tissue so that further eruption is unlikely. The mandibular third molar is the most common tooth to be impacted, which can be due to space deficiency, angulation of the third molar, ectopic position, obstruction along the pathway of eruption, follicular collision, etc. Impacted mandibular third molar (IMTM) can be symptomatic or asymptomatic. However, surgical extraction of IMTM is one of the common procedures performed by oral surgeons, which is mostly followed by postoperative sequelae like pain, swelling, and trismus causing discomfort and affecting the patient’s day-to-day activity [1]. Alveolar osteitis (AO) also termed dry socket or fibrinolytic osteitis is one of the complications that clinically presents with pain and halitosis after 48 hours which happens due to disruption of the clot, which has a 4.05% chance of occurrence [2]. Therefore, clinically it’s spotlighted the importance of the development of newer techniques to reduce postoperative sequelae [3].

Wound healing is a complex process that is associated with various cells like epithelial cells, mesenchymal cells (osteoblasts, fibroblasts), and various signaling molecules. For the regeneration of soft and hard tissue, various techniques have been used in the maxillofacial region. The growth factor plays an indispensable role in increasing mitotic cell division, initiating angiogenesis, stimulating collagen synthesis, and inducing cell differentiation [4]. Increasing platelets concentrated at the site of the wound is proven to promote more rapid and better healing, inducing osteo-induction and bone healing. Platelet concentrate applications have been used for almost more than half a century and have been modified from their predecessors to improve their biological efficiency, effectiveness, and clinical performance and to prevent side effects. Initially, in the 1970s donor plasma along with thrombin and calcium were used for the polymerization of fibrinogen which fell into major pitfalls of disease transmission [5]. Older techniques proposed by Tayapongsak and Whitman utilized the double spin technique and are time-consuming and technique-sensitive [6].

Later platelet-rich plasma (PRP) an autogenous platelet concentrate as a feasible means of growth factor delivery was developed. PRP is prepared by centrifuging entire blood samples with the addition of anticoagulant. PRP has a positive impact on improving bone density, soft-tissue repair, and pain reduction after impacted third mandibular extraction but the downside of PRP is the chances for allergic or anaphylactic reaction due to the addition of anticoagulant [7]. To prevent the flaw of anticoagulants, second-generation platelet concentrates were developed. Platelet-rich fibrin (PRF) is effective when compared with PRP in reducing postoperative pain and also prevails over the drawback of using additives like bovine thrombin or anticoagulants [8]. A study proved a lower severity of pain after PRF application in the extraction socket. PRF with chlorhexidine enhances the efficiency of PRF and reduces the chance for occupancy of dry sockets after surgical extraction of IMTM [8].

One of the modifications of the second generation of platelet concentrates is advanced platelet-rich fibrin (A-PRF) which was introduced by Dr. Choukroun. It uses lower g force for centrifugation (1500 rpm for 14 minutes) thereby resulting in higher concentrations and gradual release of growth factors than predecessor platelet concentrates which enhances postoperative wound healing [9]. Alpha granule contents of A-PRF are chemokines like eotaxins, CCL5. Eotaxin/CCL11 (eosinophil-special chemokine) and CCL5 (pro‐inflammatory chemokines that are chemotactic for T cells and leukocytes) are secreted at a higher rate by A-PRF [10]. Vascular endothelial growth factor and platelet-derived growth factor favor the chemotaxis of macrophages and the migration of fibroblasts to stimulate angiogenesis. PDGF-AB, thrombospondin-1 (TSP-1), tumor necrosis factor B-1 (TGF-1) interleukins (IL-1B, IL-6, and IL-4), and tumor necrosis factor o (TNF o) are found in significant quantities in A-PRF [11]. Leukocytes stimulate pro and anti-inflammatory mediators, VEGF and TGF B-1. A-PRF has enhanced mechanical properties, cell viability, and synthesis of alkaline phosphatase [12]. The porous structure of A-PRF aids in better penetration of endothelial cells to stimulate rapid angiogenesis [13].

C-reactive protein concentration is a marker of inflammation that is associated with postoperative healing which returns to normal levels after 7 days. A-PRF allows faster reduction of C-reactive protein (CRP) concentration and reduces chances for occupancy of dry sockets [14]. A-PRF preparations not only scaffold but also reserve growth factor, which mimics the physiology and immunology of wound healing. Higher levels of proliferation and migration of fibroblasts, mRNA, and collagen levels have also been reported with A-PRF [15]. It has been reported that a higher total protein accumulation at a duration of 10 days happens in A-PRF when compared with their predecessor (PRP or PRF) [16,17].

Hence, this study aimed to assess the efficiency of A-PRF in wound healing after surgical extraction of the IMTM and its impact on the postoperative sequelae like pain.

## Materials and methods

Study design and setting

Patients reported to the Department of Oral and Maxillofacial Surgery, Saveetha Dental College and Hospital from January 2023 to May 2023 for management of IMTM were included in the study. Ethical clearance was obtained from the institutional human ethical committee with the institutional review board number - IHEC/SDC/OMFS-2107/23/193 and the study was conducted after obtaining informed consent from all the patients.

Inclusion criteria

Clinically healthy patients without any pre-existing periodontitis who have been diagnosed with asymptomatic Pell Gregory’s class II IMTMs in the ages of 18 years and above were included in this prospective clinical study.

Exclusion criteria

IMTM associated with pericoronitis, periodontitis or with any pathology or temporomandibular joint (TMJ) disorder, smokers, patients with any bleeding disorder, systemic illness, and compromised patients were excluded from this study.

Surgical procedure

**Figure 1:**
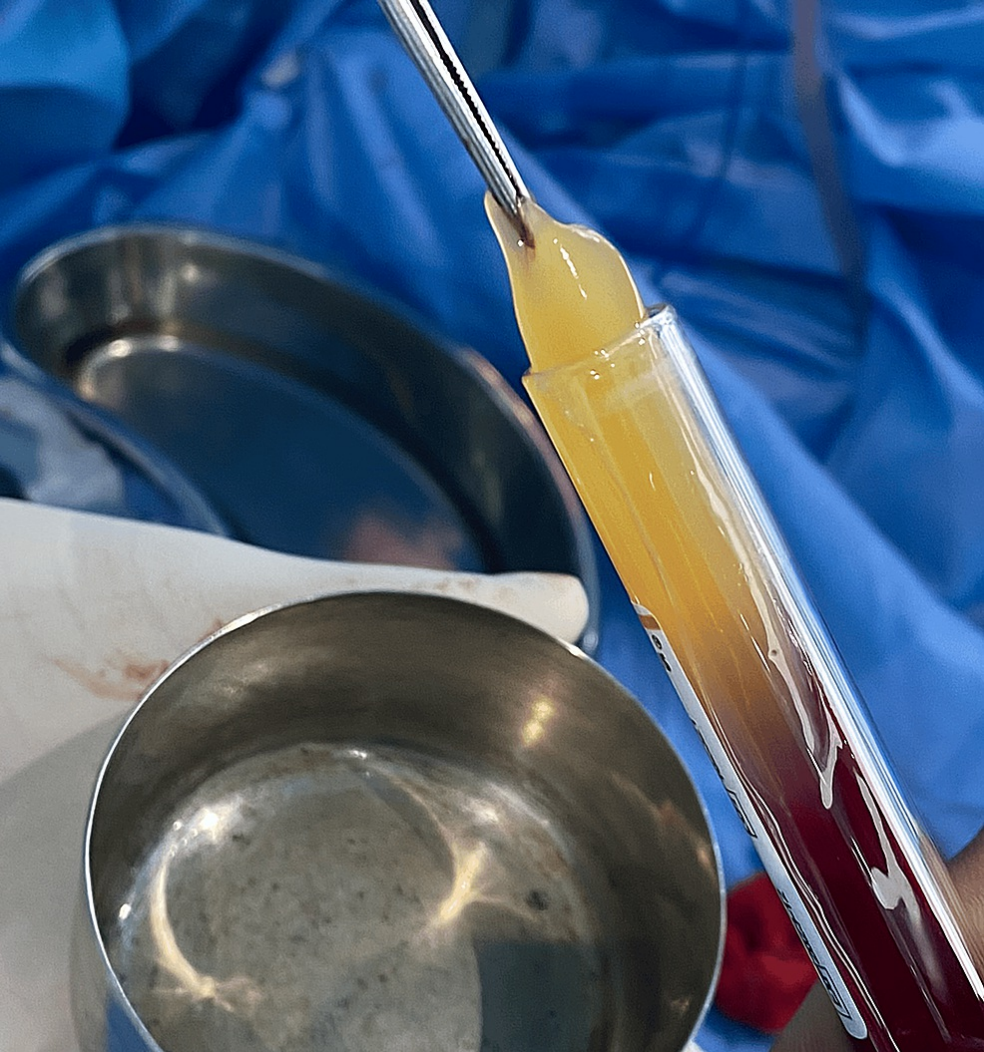
Advanced platelet-rich fibrin extract.

The study consisted of 30 individuals with 15 samples in each group allocated using a simple random sampling technique. An opaque envelope was used for allocation. Surgical removal of impacted teeth was done under local anesthesia by the same surgeon and assessments were done by an investigator in all the groups. Double blinding was followed in this study as neither the patient nor the assessor was aware of the material used in the groups. All surgical extraction of IMTM was done by the same surgeon. Under aseptic conditions, the inferior alveolar nerve block was given with 2% lignocaine with 1:200000 adrenaline, modified ward's was incision placed, full-thickness mucoperiosteal flap was elevated, and buccal, mesial, and distal bone guttering was done accordingly. A tooth split was done if needed and the tooth was elevated and extracted. The extracted socket was thoroughly cleaned to remove the granulation tissue and bony spicules. Based on the A-PRF preparation protocol, the required blood sample of 10 ml was taken from the antecubital vein [4]. Collected blood samples were placed in sterile test tubes with no addition of anticoagulant. Centrifugation speed was set at 1500 rpm (230 g) for a duration of 14 minutes at room temperature. The fibrin-rich clot was extracted carefully after the removal of the overlying red blood cell fraction (Figure 1).

The extracted A-PRF’s size was alerted to fit into the extraction socket at the bony crest level. After application of A-PRF, suturing was done with 4-0 braided silk sutures. A-PRF was filled in extraction sockets and closure was done with 4-0 braided silk sutures in the study (intervention) group and the control group was primarily closed with 4-0 braided silk with no additional intervention. Primary closure with 4-0 silk sutures was done in a similar manner for all our participants in our study to avoid bias. Postoperatively antibiotics (amoxicillin 500 mg) and analgesics (combination of paracetamol and aceclofenac) were prescribed to both groups for 5 days and no mouthwash was prescribed.

Visual analog scale (VAS) is usually presented as a 100-mm horizontal line with the absolute value of pain from 0-10 on the scale and grouped into three categories: 1-4 (mild pain), 5 to 6 (moderate pain), and 7-10 (severe pain) [4]. The lower the pain value, the lesser the pain perception, which is measured preoperatively before the administration of the local anesthesia and, on the 3rd, and 7th postoperative days. A modified laundry scale was used for clinical wound healing assessment, and no radiographic evaluation was done. Tissue color, bleeding on probing (Williams periodontal probe was used), presence of granulation tissue, and nature of incision margin were taken into consideration and then categorized into either very poor, poor, good, very good, or excellent categories. The higher the criteria better the wound healing and the wound healing assessment was done on the 3rd and 7th postoperative days.

Statistical analysis

SPSS for Windows (SPSS version 22.0, IBM Corp., Armonk, NY) was used for data analysis. Postoperative pain and wound healing were compared between the groups using the Chi-square test. The data was presented using graphs and tables. The level of significance was set at p < 0.05.

## Results

The mean age of the 30 participants was 25.67 ± 2.4 years. Nineteen patients were males, and 11 patients were females. Pain assessment between the groups using VAS scores is depicted in Table 1. On the 3rd day in the intervention group, it was found that 14 participants presented with mild pain and one participant presented with moderate pain, and in the control group 12 participants presented with mild pain, and three participants with moderate pain. This pain difference between groups was statistically not significant (P = 0.59). On the 7th day in the intervention group, four participants presented with no pain and 11 participants with mild pain, and in the control group one participant presented with no pain, and 14 participants presented with mild pain. This pain difference between groups was statistically not significant (P = 0.33).

**Table 1:** Comparison of pain among the participants between the groups at baseline, 3rd and 7th postoperative days. N: number; %: percentage; NS: not significant (chi-square test)

Groups	No Pain	Mild Pain	P-value
Baseline	N (%)	N (%)	
Intervention (n=15)	3 (20)	12 (80)	P = 0.99
Control (n=15)	4 (26.7)	11 (73.3)	NS
	Mild Pain	Moderate	P-value
3rd Day	N (%)	N (%)	
Intervention (n=15)	14 (93.3)	1 (6.7)	P = 0.59
Control (n=15)	12 (80)	3 (20)	NS
	No Pain	Mild Pain	P-value
7th Day	N (%)	N (%)	
Intervention (n=15)	4 (26.7)	11 (73.3)	P = 0.33
Control (n=15)	1 (6.7)	14 (93.3)	NS

Wound healing assessed between the groups is depicted in Table 2. On the 3rd day in the intervention group, one participant presented with poor wound healing, nine participants with good wound healing, and five participants presented with very good wound healing. In the control group, five participants presented with poor wound healing, nine participants presented with good wound healing, and one participant presented with very good wound healing. This difference in wound healing distribution was not statistically significant (P = 0.17). On the 7th day in the intervention group, one participant presented with good wound healing, seven participants presented with very good wound healing, and six participants presented with excellent wound healing. In the control group, one participant presented with poor wound healing, eight participants presented with good wound healing, and five participants presented with very good wound healing. This difference in wound healing distribution was statistically significant (P = 0.01) and the intervention group (A-PRF) exhibited better wound healing properties than the control group.

**Table 2:** Comparison of wound healing among the participants between the groups on 3rd and 7th postoperative days. N: number; %: percentage; NS: not significant **statistically significant (chi-square test)

3rd Day	Poor	Good	Very Good	Excellent	Total	P-value
Groups	N (%)	N (%)	N (%)	N (%)		
Intervention	1 (6.7)	9 (60)	5 (33.3)	0	15	P = 0.17
Control	5 (33.3)	9 (60)	1 (6.7)	0	15	NS
7th Day	Poor	Good	Very Good	Excellent	Total	P-value
Groups	N (%)	N (%)	N (%)	N (%)		
Intervention	0	1 (6.7)	7 (46.7)	7 (46.7)	15	P = 0.01**
Control	1 (6.7)	8 (53.3)	5 (33.3)	1 (6.7)	15	

## Discussion

The effectiveness of A-PRF in reducing postoperative sequelae after surgical extraction of IMTM was assessed in this study. To reduce bias, patients with Pell and Gregory’s class II IMTMs and with no history of comorbidities and systemic illnesses were included in the study and all patients were operated by the same surgeon. Our study consisted of 30 participants (19 males and 11 females), and the role of gender in wound healing was not assessed. Njokanma et al. in their study showed that females exhibited 7.04% more bone formation in the apical third than the males [3]. However, these findings were statistically not significant which can be attributed to the limited sample size. Similar results were shown in the study by Areewong et al. [18].

A-PRF due to its anti-inflammatory properties can significantly reduce pain, swelling, and trismus after lower third molar surgery. A study reported that the harmful effects of inflammation are reduced by the immune regulatory activity of A-PRF [19]. A systematic review included three randomized controlled trials (RCTs) that used A-PRF and showed reduced postoperative pain [10]. Cayman et al. also found a similar result in their study which was conducted on 27 patients to compare the effectiveness of A-PRF and L-PRF and reported a significant reduction in pain. This in turn reduced the quantity of analgesic medications consumed postoperatively [20]. However, our study results showed no significant difference in VAS scores on the 3rd and 7th postoperative days between the case and control groups. Similar results were found in another study which included 75 patients and they concluded that A-PRF has no positive effect on postoperative sequelae [21].

A-PRF has better soft-tissue healing due to the release of growth factors for up to 8-10 days. Similar results were found by Kobayashi et al. in their comparative study in which A-PRF released higher total protein which accumulated for up to 10 days [7]. Choukroun et al. found that A-LRF mimics the physiology and immunology of wound healing by their higher concentration of growth factors, and also porous nature of A-PRF allows better penetration of endothelial cells which in turn induces angiogenesis [22]. Several studies reported that A-PRF significantly improved soft-tissue healing compared to natural wound healing [23,24]. In our study, on the 7th postoperative day, statistically significant improvement in wound healing was observed in the A-PRF group compared to the control group. On the contrary, Zahid et al. in their study included 10 patients and found no significant difference in gingival recessions, clinical attachment loss, and pocket depth between the study and control groups [25].

Limitations of the study

As pain perception and wound healing vary from person to person, further split-mouth studies with larger sample sizes are needed to formulate protocols for clinical usage and for assessing the clinical efficiency of A-PRF.

## Conclusions

A-PRF is a simple and effective method of reducing postoperative sequelae which promotes wound healing after surgical extraction of IMTM. A-PRF has an advantage over its platelet concentrate predecessors with fewer chances of allergic and anaphylactic reactions. However, further split-mouth studies with larger samples are needed to formulate clinical protocols and to assess the clinical efficiency of A-PRF.
